# Supplementary material for: Patterns of Intron Gain and Loss in Fungi
Source: PLoS Biol. 2004 Nov 30;2(12):e422. doi: 10.1371/journal.pbio.0020422 (PMC532390; doi:10.1371/journal.pbio.0020422)
Supplement: Table S1 — Also available at http://genes.mit.edu/NielsenEtAl/. (4.3 MB ZIP). [file pbio.0020422.st001.zip › NielsenEtAl/html/164.html]

AN1261.1.NCU07928.1.MG05157.1.FG05613.1


```
 CLUSTAL W (1.82) Multiple Sequence Alignments - Introns Inserted


Sequence 1: NCU07928.1	227 aa
Sequence 2: FG05613.1	221 aa
Sequence 3: MG05157.1	229 aa
Sequence 4: AN1261.1	248 aa
Alignment Length: 257 aa
Number Identitical Residues: 62 aa
Alignment Score (without introns) 3528


MG05157.1 	MPSDPFCISFDATFDGHYEKICSNVEVQVSAISTPERLETPEDSED--------------
NCU07928.1	MAISQGPVGVAMASHST----PVHPEKTSSIVSENNIQTTPTELPD--------------
FG05613.1 	MAN----VDVQTAPG-------AALDIFSKNVGQVDIRDISSTLTP--------------
AN1261.1  	MTAASVTTAALVATDGG----PTLPQAQLSAIPEPPAMPTAVGAPTGFHVEFQSAPVLNP
          	*.          :  .         :   . :        .     .   . .::.  ..

MG05157.1 	--------DTRYLLVSPYTEKPHLLDLDSVDVENQLLAQALVYMNNVRDDFRTASYQDSF
NCU07928.1	--------KERYIVVSPYIEQQHLLDLTTLDPENQILALALSQLTCLREDYATAPYRDTF
FG05613.1 	--------SDRYLVQSPYTEQEHLLDLETLDNENTILAHALGKFRALRDDYATAPYTESF
AN1261.1  	LEDEPSKAQGNLLIESPYITREHLLDLSTLDTQNRLLAQALTTLTPVRPDYATAPYTESF
          	 ....:.:. . :: ***  : ***** ::* :* :** **  :  :* *: **.* ::*

MG05157.1 	NWHEIIQHLRRLNQRQGHDWKETSFYIVAFRSQIKPDA--DYSHLVELDRYAHAEATASG
NCU07928.1	NWDQIIEHVRALARKRNYTWKETSFFVVAFRSQVFESTVENYADLGELDKAAHAEATASG
FG05613.1 	NWPEVMEEVKHLALESGKGFKETSFYIVAFRSRIKQTT--EYADLGRLDKGAHAEAVASG
AN1261.1  	NWDFVFLKFYSLAAKEGYSLSRQSFYVVVFRSVLQANA--DGDRLLLLDERSHAEAVSSG
          	**  :: ..  *  . .   .. **::*.*** :   :  :   *  **. :****.:**

MG05157.1 	GFLK2YWFGEYPDEQGHNLATC~VWTSRQHARRGNVGPAHREAMKAA-SLYAWHELERYE
NCU07928.1	GFLK2YWFGT-PNEERRNLATC~IWRSPEDAKKGGTGPAHRKAAGATRSLYSFWHIDRHR
FG05613.1 	GFLK2YWFGE-PDSVLANLATC~IWRSREDAKNGGTGPAHRKAAGATRSMYAFWKIDQHR
AN1261.1  	GLLK~YWFGV-KNGRRENLATC1LWRNREDARAGGTGPWHAQARAAAREMYEKIEFTTLE
          	*:** ****   :    ***** :* . :.*: *..** * :*  *: .:*   .:   .

MG05157.1 	LTVRAGVEGWSLTRLG---
NCU07928.1	LTIRDNVESWDLTEWN---
FG05613.1 	LIVRDNAEDWEIIPWEDSA
AN1261.1  	LVVGEKAGEWEFREWKH--
          	* :   .  *.:
```
